# Supplementary material for: Alpha-1 Antitrypsin Reduces Disease Progression in a Mouse Model of Charcot-Marie-Tooth Type 1A: A Role for Decreased Inflammation and ADAM-17 Inhibition
Source: Int J Mol Sci. 2022 Jul 3;23(13):7405. doi: 10.3390/ijms23137405 (PMC9266995; doi:10.3390/ijms23137405)
Supplement: Supplementary file 1 [file ijms-23-07405-s001.zip › Supplementary figures plus legend_Revised 27.6.2022.pdf]

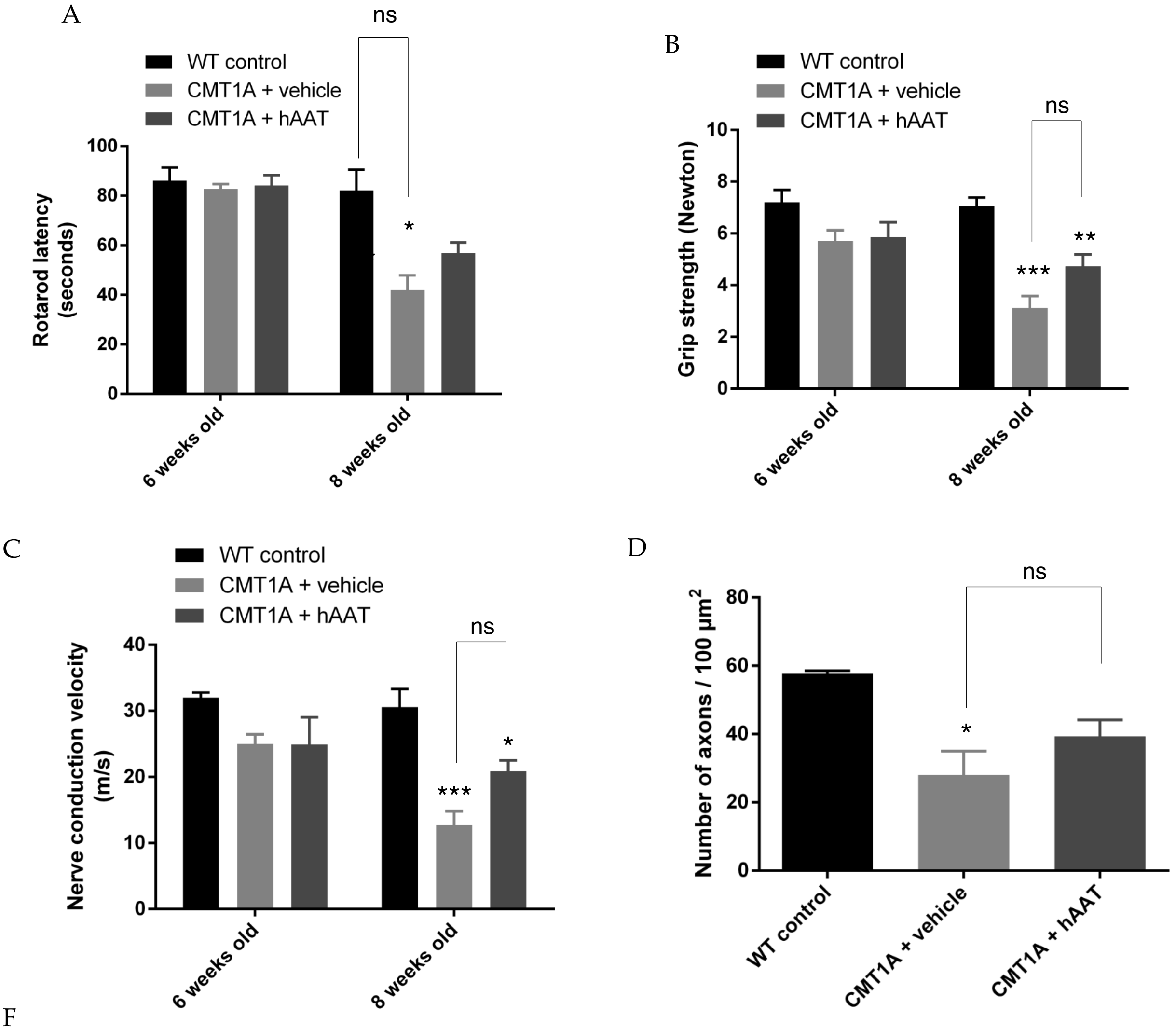

F

	Day 1	Day 8	Day 14	Day 29
WT control	2.60 ± 0.05	2.89 ± 0.14	2.80 ± 0.187	2.80 ± 0.187
CMT1A + vehicle	2.76 ± 0.17	3.99 ± 0.11**	5.80 ± 0.175***	11.81 ± 0.897***
CMT1A + AAT	2.94 ± 0.17	3.70 ± 0.41	5.15 ± 0.393**	10.67 ± 0.833***

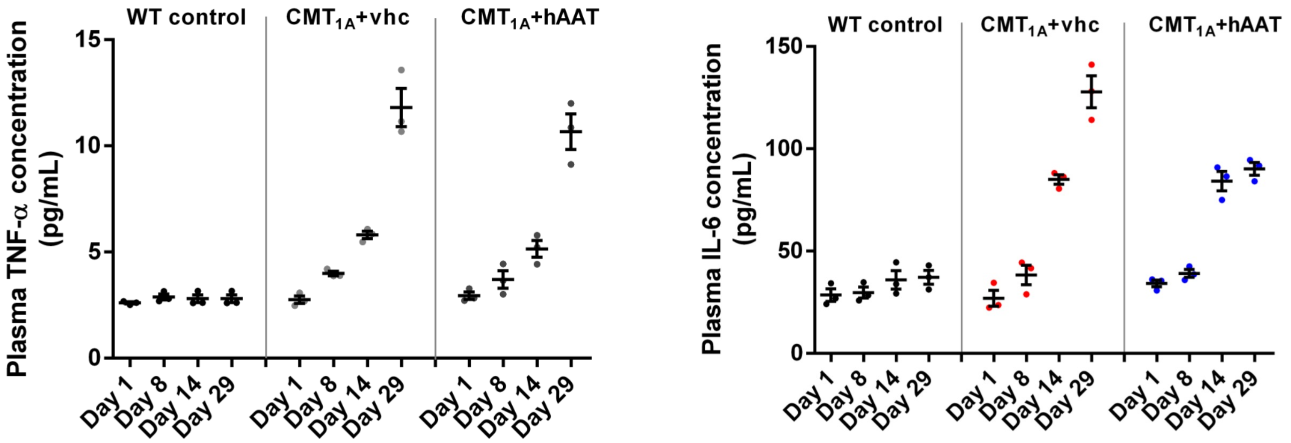

Figure S1. Rotarod Latency (A) Grip strength (B)At 8 weeks of age, CMT1A+ vehicle mice exhibited impaired neuromuscular performance relative to the wild type animals. CMT1A+hAAT mice presented a positive but not significant tendency in both neuromuscular test compared to CMT1A+vehicle. Nerve Conduction velocity (C) positive but not significant trend was observed in CMT1A mice treated with hAAT for this electric measurement. Number of axons (D) Slight increase of the total number of axons per surface was observed in the CMT+hAAT compared to CMT1A+vehicle. (F) Plasma TNF-α and IL-6 ELISA measurements.
Mean ± SEM, n=3 per group; 2-way ANOVA with repeated measures and Bonferroni t-test *; **;***: p<0.05; p<0.01; p<0.001 vs WT control. WT: wild type, not significative (ns) vs CMT1A+vehicle. For TNF-α and IL-6 concentration Mean ± SEM, Student t-test *; **; ***: p<0.05; p<0.01; p<0.001 vs WT control at this timepoint. For IL-6 concentration value see also Table 3 in the main text.
For all the experiments n=3 mice per group.

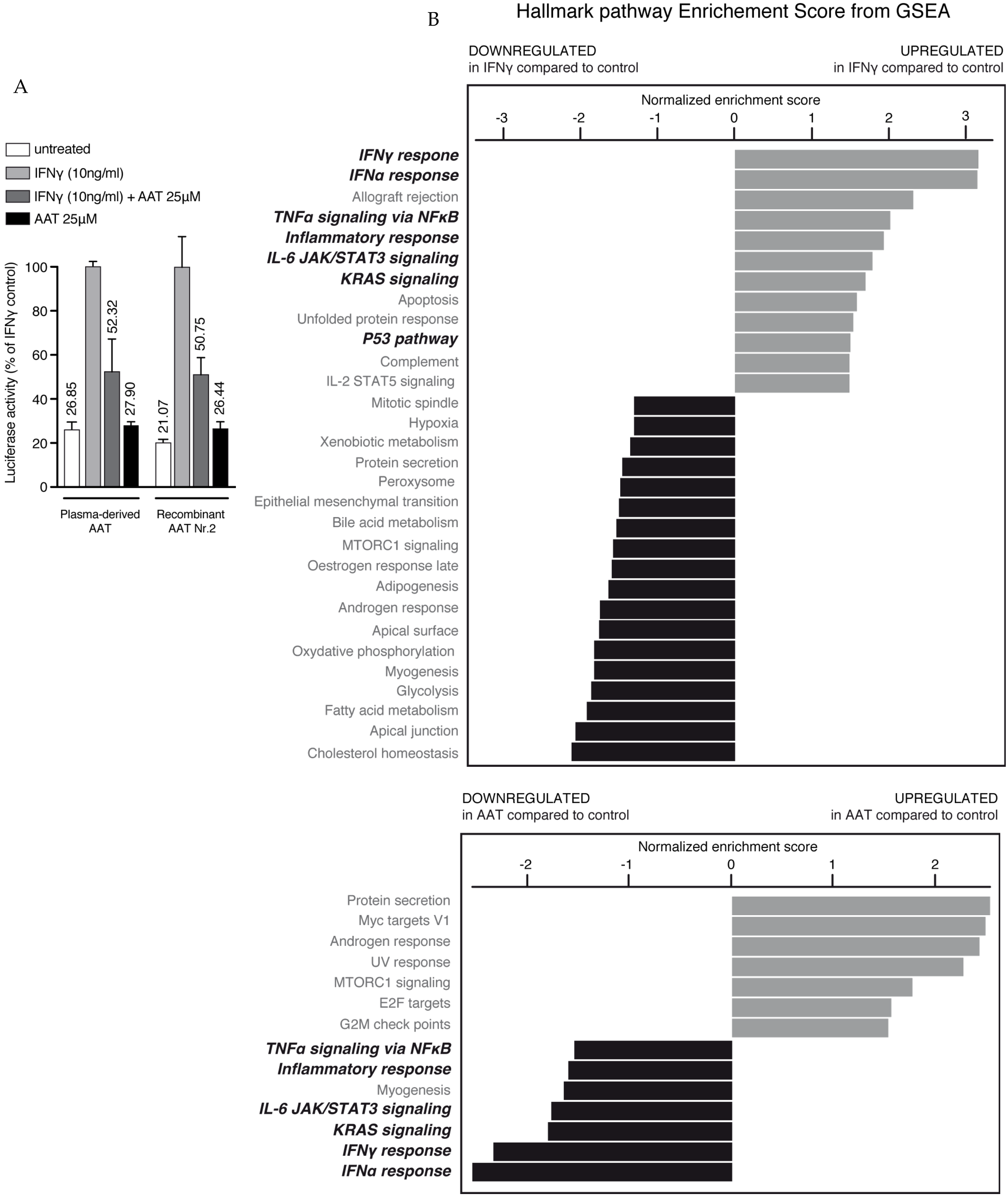

Figure S2. MHCII luciferase activation after IFNγ induction followed by plasma hAAT and recombinant AAT (rhAAT) (A). Luciferase activity is normalized over cell viability. Bars represented percentage of control for all conditions. Gene Set Enrichment Analysis (GSEA) of RNAseq data from plasma and rhAAT treated cells (B). Upregulated gene families (grey bars) and downregulated ones (black bars) are shown according to normalized enrichment score. The inflammatory profile induced by IFNγ was confirmed by the upregulation of several processes related to inflammation (bold italic), AAT in absence or presence (see Figure 9) of inflammation was able to downregulate some of these pathways (bold italic).

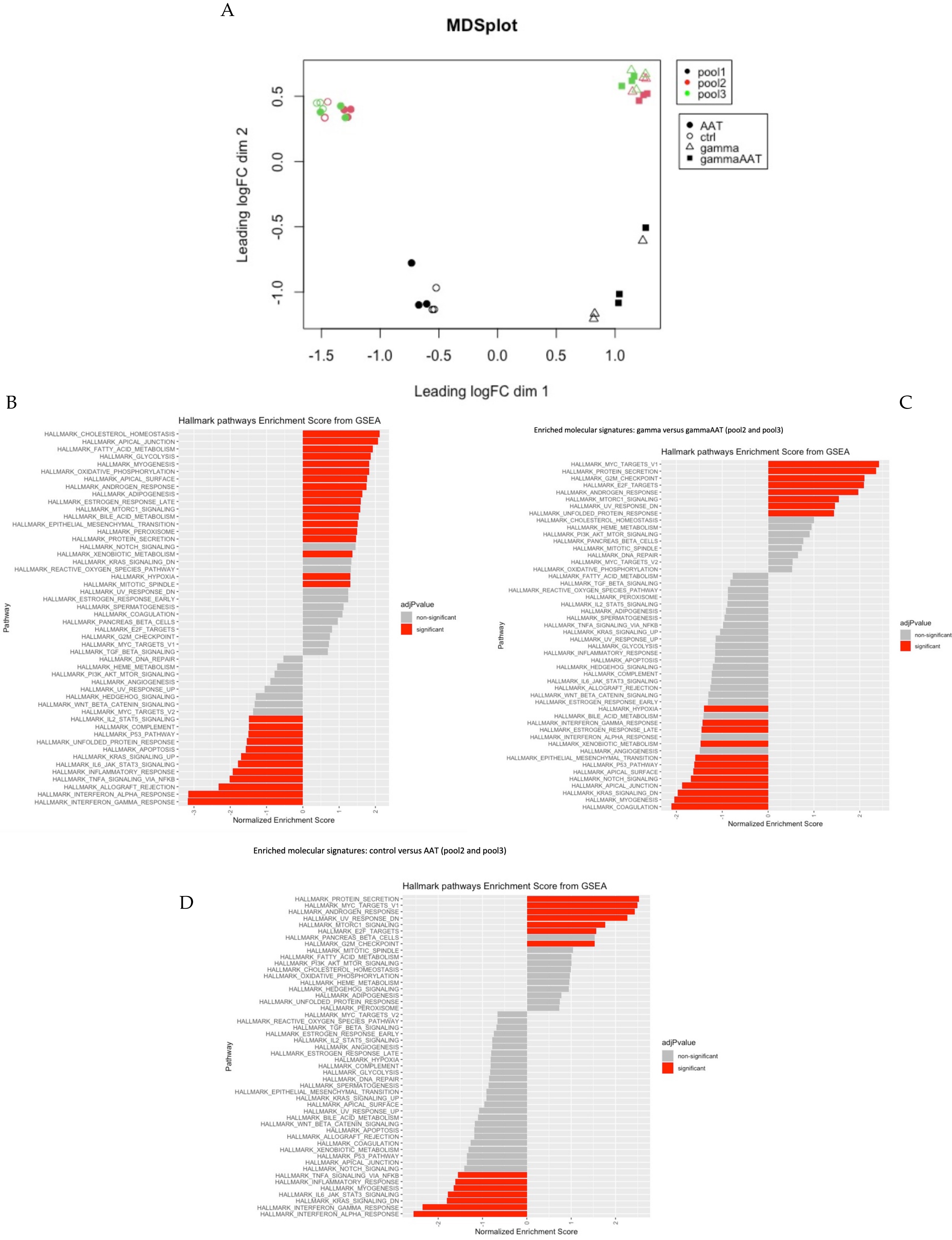

Figure S3. The MDS plot (multi-dimensional scaling) of the samples is shown (A). The MDS plot gives an indication of the similarity, based on the fold changes between all the pairs of samples. Enriched molecular signatures: control versus gamma (pool2 and pool3) (B). Enriched molecular signatures: control versus AAT (pool2 and pool3) (C). Enriched molecular signatures: gamma (IFNγ) versus gammaAAT (IFNγ + AAT) (pool2 and pool3) (D).
A negative score (NES) means that the genes belonging to that gene set are mostly down regulated in the comparison tested and a positive NES that they are upregulated in the tested comparison.

Antigen presentation

Top Gene	p-value	FC	p-value	FC
	Up in inflammation		DOWN (Inflammation+AAT)	
**CD74**	4.15E-14	75.81		
**LAMP3**	9.39E-12	7.94		
**TAP1**	5.99E-11	6.12		
**PSMB9**	**5.73E-11**	**5.96**	**3.11E-02**	**-1.23**
**HLA-DOB**	**5.11E-04**	**4.48**	**6.82E-03**	**-4.49**
**PSMB8**	5.18E-11	4.22		
HLA-DRA	***4.99E-10***	***4.76***	***4.68E-02***	**-2.22**
**SOCS1**	1.21E-05	3.99		
**TAP2**	8.97E-13	3.54		
**NCF2**	2.57E-05	2.88		
**CTSS**	2.29E-05	3.71		
**HLA-DPA1**	8.31E-09	3.22		
**BATF3**	1.45E-06	2.55		
**NFKB1**	6.21E-10	2.41		
**IL1B**	**5.54E-04**	**2.08**	**1.11E-03**	**-2.07**
**FYN**	2.18E-07	1.80		
**BCL10**	3.21E-05	1.65		
**MALT1**	1.75E-04	1.62		
	DOWN in inflammation		UP in inflammation+AAT	
**TUBA1A**	2.29E-12	-5.00		
**KIF20A**	7.29E-12	-3.75		
**CALR**	**6.15E-11**	**-3.53**	**4.74E-04**	**1.35**
**PYCARD**	**6.92E-03**	**-2.86**	**1.53E-02**	**2.20**
**CDC20**	1.05E-08	-2.80		
Other Gene	p-value	FC	p-value	FC
	Down in inflammation		UP in inflammation+AAT	
**NFATC2**	<0.05	-1.37	<0.05	1.63
**MRC2**	<0.05	-1.61	<0.05	1.47
**CTSD**	<0.05	-1.34	<0.05	1.47
**HLA-DPB1**	<0.05	-1.23	<0.05	1.31
**TAPBP**	<0.05	-1.90	<0.05	1.23
**SOCS3**	<0.05	-1.99	<0.05	1.20
**CTSA**	<0.05	-1.31	<0.05	1.17
	Regulated in inflammation		Regulated in AAT	
**NFATC1**	<0.05	-1.29	<0.05	1.44
**TRAF6**	<0.05	1.08	<0.05	-1.26

Figure S4. Gene related to antigen presentation. Inflammatory top gene (FC>2; p-value<0.05; left column), were defined by differential expression between untreated and IFNγ-treated cells (inflammation; middle column). Top genes that were significantly and oppositely regulated by AAT treatment are highlighted (right column; underline).

Cytokine signaling				
Top Gene	p-value	FC	p-value	FC
	UP in inflammation		DOWN in inflammation+AAT	
**IL6**	3.53E-10	17.139		
**TNFSF10**	2.53E-07	10.595		
**TRAF1**	4.39E-07	7.789		
**CCL5**	5.17E-09	7.540		
**ATF3**	**6.86E-13**	**7.218**	**1.85E-03**	**-1.26**
**PSMB9**	**6.09E-10**	**5.963**	**3.11E-02**	**-1.23**
**CEBPB**	2.57E-10	5.808		
**CSF1**	3.52E-09	5.455		
**PSMB8**	1.63E-10	4.219		
**IL23A**	6.49E-03	4.218		
**STAT1**	2.04E-09	4.110		
**IL15RA**	4.63E-07	3.660		
**FGF2**	**1.66E-06**	**3.608**	**9.13E-03**	**-1.57**
**CXCL2**	6.42E-05	3.467		
**IL21R**	1.16E-05	3.235		
**CEBPG**	3.19E-09	2.909		
**CXCL1**	**1.36E-04**	**2.82**	**7.22E-03**	**-2.09**
**CASP1**	1.07E-05	2.740		
**TNFRSF9**	**2.39E-04**	**2.434**	**1.23E-03**	**-2.35**
**CXCL5**	**5.07E-05**	**2.36**	**5.98E-03**	**-2.15**
**IL1B**	**2.14E-03**	**2.08**	**1.11E-03**	**-2.07**
	DOWN in inflammation		UP in inflammation+AAT	
**FSCN1**	**9.42E-08**	**-6.312**	**8.09E-04**	**1.48**
**CCL2**	2.95E-03	-6.086		
**IL4I1**	**3.22E-03**	**-4.19**	**3.22E-03**	**1.95**
**IL27RA**	**2.22E-07**	**-3.848**	**2.67E-02**	**1.36**
**STAT5A**	**7.66E-04**	**-3.53**	**9.78E-03**	**1.45**
**CEBPD**	5.07E-05	-3.102		
**EGF**	6.13E-03	-2.973		
**FGF18**	2.11E-05	-2.754		
**IL6R**	6.00E-08	-2.630		
**IL1R1**	**1.27E-07**	**-2.566**	**3.94E-02**	**1.18**
**JAK3**	**3.03E-08**	**-2.43**	**3.20E-03**	**1.43**
**RASAL1**	**1.36E-06**	**-2.34**	**4.65E-03**	**1.52**
Other Gene	p-value	FC	p-value	FC
	UP in inflammation		DOWN in inflammation+AAT	
**IL18**	<0.05	1.83	<0.05	-1.75
**NFKBIZ**	<0.05	1.94	<0.05	-1.37
**ANXA1**	<0.05	1.17	<0.05	-1.33
**HBEGF**	<0.05	1.36	<0.05	-1.30
**DUSP6**	<0.05	1.96	<0.05	-1.26
	DOWN in inflammation		UP in inflammation+AAT	
**CX3CL1**	<0.05	-1.38	<0.05	1.61
**MAP2K2**	<0.05	-1.28	<0.05	1.51

Figure S5. Genes related to cytokines signaling. Analysis was performed as described in Figure S4.
